# Proteomics analysis reveals differential acclimation of coastal and oceanic *Synechococcus* to climate warming and iron limitation

**DOI:** 10.3389/fmicb.2024.1323499

**Published:** 2024-02-20

**Authors:** Cara Schiksnis, Min Xu, Mak A. Saito, Matthew McIlvin, Dawn Moran, Xiaopeng Bian, Seth G. John, Qiang Zheng, Nina Yang, Feixue Fu, David A. Hutchins

**Affiliations:** ^1^Marine and Environmental Biology, University of Southern California, Los Angeles, CA, United States; ^2^State Key Laboratory of Marine Resource Utilization in South China Sea, Hainan University, Haikou, China; ^3^Marine Chemistry and Geochemistry Department, Woods Hole Oceanographic Institution, Woods Hole, MA, United States; ^4^State Key Laboratory of Marine Environmental Science, Institute of Marine Microbes and Ecospheres, Xiamen University, Xiamen, China; ^5^Biology Department, Woods Hole Oceanographic Institution, Woods Hole, MA, United States; ^6^Marine Policy Center, Woods Hole Oceanographic Institution, Woods Hole, MA, United States

**Keywords:** climate change, *Synechococcus*, iron limitation, ocean warming, proteomics, interactive effects

## Abstract

In many oceanic regions, anthropogenic warming will coincide with iron (Fe) limitation. Interactive effects between warming and Fe limitation on phytoplankton physiology and biochemical function are likely, as temperature and Fe availability affect many of the same essential cellular pathways. However, we lack a clear understanding of how globally significant phytoplankton such as the picocyanobacteria *Synechococcus* will respond to these co-occurring stressors, and what underlying molecular mechanisms will drive this response. Moreover, ecotype-specific adaptations can lead to nuanced differences in responses between strains. In this study, *Synechococcus* isolates YX04-1 (oceanic) and XM-24 (coastal) from the South China Sea were acclimated to Fe limitation at two temperatures, and their physiological and proteomic responses were compared. Both strains exhibited reduced growth due to warming and Fe limitation. However, coastal XM-24 maintained relatively higher growth rates in response to warming under replete Fe, while its growth was notably more compromised under Fe limitation at both temperatures compared with YX04-1. In response to concurrent heat and Fe stress, oceanic YX04-1 was better able to adjust its photosynthetic proteins and minimize the generation of reactive oxygen species while reducing proteome Fe demand. Its intricate proteomic response likely enabled oceanic YX04-1 to mitigate some of the negative impact of warming on its growth during Fe limitation. Our study highlights how ecologically-shaped adaptations in *Synechococcus* strains even from proximate oceanic regions can lead to differing physiological and proteomic responses to these climate stressors.

## 1 Introduction

The ocean buffers fossil fuel-driven increases in temperature by absorbing excess atmospheric heat (Falkowski and Raven, 2007; Zanna et al., 2019). While this has overall helped to decelerate the rampant pace of anthropogenic climate change, it is also bringing about novel consequences for the ocean’s physiochemical and biological systems (Doney et al., 2012; Hutchins and Fu, 2017). Sea surface temperature and iron (Fe) limitation are two primary controls on phytoplankton growth and distribution that will likely be enhanced simultaneously by climate change in some regions of the ocean (Hutchins and Boyd, 2016; Hutchins and Fu, 2017). Consequently, the distribution and abundance of dominant phytoplankton groups may be considerably altered in the future.

*Synechococcus*, a genus of unicellular cyanobacteria, contributes significantly to primary productivity throughout most of the global surface ocean (Flombaum et al., 2013). Members of the *Synechococcus* genus are widespread in part due to their vast genetic and physiological diversity that allows them to inhabit a range of marine environments. A high number of diverse subgroups (Farrant et al., 2016), termed “ecotypes,” are adapted to the specific conditions of their ecological niche, including temperature and nutrient availability (Ahlgren and Rocap, 2012; Sohm et al., 2016). As climate change progresses, the ecological success of *Synechococcus* ecotypes will depend on their ability to respond to increasingly stressful conditions such as concurrent sea surface warming (Mackey et al., 2013; Six et al., 2021) and Fe limitation (Ahlgren et al., 2020; Gilbert et al., 2022).

On its own, temperature controls phytoplankton growth and metabolism in a relatively predictable way. Rates of photosynthesis and respiration typically increase until an optimum growth temperature is reached, and then rapidly decline as deleterious effects of heat stress take place, including protein denaturation and oxidative damage (Went, 1953; Falk et al., 1996; Hutchins and Fu, 2017). As temperature is a central driver of many cellular processes, phytoplankton have adapted to the thermal range of their environmental niche, and thermal physiology of *Synechococcus* ecotypes has been found to correlate with location (Zwirglmaier et al., 2008; Thomas et al., 2012; Pittera et al., 2014). However, despite a developed understanding of the effect of temperature on phytoplankton growth, metabolism, and distribution, a central temperature regulatory response system in *Synechococcus* is not well-constrained. Some models have predicted that ocean warming will increase the abundance and distribution of *Synechococcus* (Morán et al., 2010; Flombaum et al., 2013), but a limited understanding of how thermal acclimation is constrained by Fe availability clouds these projections (Tagliabue et al., 2020).

The essential micronutrient Fe is required by all phytoplankton for the electron transport chains of photosynthesis and respiration, and as a cofactor in enzymes involved in nutrient uptake, chlorophyll biosynthesis, and oxidative stress management (Raven et al., 1999; Scanlan et al., 2009). Because of the high requirement for Fe in photoautotrophic growth, primary productivity is limited by low Fe concentrations across more than one third of the surface ocean (Martin, 1990; Moore et al., 2013). While much of the open ocean is chronically Fe limited, input from dust, upwelling, or riverine sources alleviates Fe limitation either seasonally or entirely in some regions. Overall, Fe availability typically declines along a gradient from coastal to offshore habitats, though coastal Fe concentrations can fluctuate throughout the year (Tagliabue et al., 2017; Twining et al., 2021; Zhang et al., 2022).

Increasing evidence suggests the importance of this complex Fe landscape in shaping *Synechococcus* ecotype diversity (Mackey et al., 2015; Sohm et al., 2016; Ahlgren et al., 2020; Gilbert et al., 2022). Physiological effects of Fe limitation include reductions in growth rates, cell size, and photosynthetic efficiency (Behrenfeld and Kolber, 1999; Jacq et al., 2014; Mackey et al., 2015). Key molecular responses, such as the ability to adjust photosynthetic machinery, the use of Fe uptake mechanisms, and an overall reduction in Fe-containing proteins, help support cyanobacterial growth under low Fe (Webb et al., 2001; Mackey et al., 2015; Ahlgren et al., 2020; Yong et al., 2022). Additionally, like heat stress, Fe limitation escalates the production of harmful reactive oxygen species (ROS) when the supply of electrons generated by the light-driven reactions of photosynthesis exceeds their demand (Michel and Pistorius, 2004; Latifi et al., 2005).

Despite the impact of temperature and Fe on *Synechococcus* fitness, genetic diversity, and distribution, an understanding of the responses of different ecotypes to these concurrent stressors remains insufficient (Hutchins and Boyd, 2016; Hutchins and Tagliabue, in review). Recent studies have focused on the proteomic response of *Synechococcus* to warming in relation to macronutrients such as nitrogen and phosphorus (Li et al., 2019; Dedman et al., 2023). However, as warming-driven stratification is predicted to restrict transport of dissolved Fe to the surface ocean in some regions, understanding how *Synechococcus* will cope with simultaneous warming and Fe limitation is increasingly pertinent, especially since these two stressors affect many of the same cellular processes (Polovina et al., 2008; Hutchins and Fu, 2017). A study on Antarctic diatoms (Jabre and Bertrand, 2020) and two studies on nitrogen-fixing cyanobacteria (Jiang et al., 2018; Yang et al., 2021) observed warming-enhanced growth or metabolic functioning of Fe limited cells. As temperature and Fe availability are shifting at the same time throughout the ocean, it is challenging to predict the subsequent interactive effects on growth and the underlying molecular mechanisms without experimental data.

To understand the impact of simultaneous warming and Fe limitation on diverse *Synechococcus* ecotypes, we assessed the physiological and proteomic acclimation responses of oceanic and coastal *Synechococcus* from the South China Sea. Oceanic strain YX04-1 was isolated from the permanently stratified South China Sea basin and is a member of the abundant, oligotrophic *Synechococcus* clade II (Du et al., 2013). Coastal strain XM-24 was isolated from the high-nutrient Xiamen estuary region, which experiences a more dynamic Fe and temperature profile. This strain belongs to clade CB5 in subcluster 5.2, members of which are typically found in river-influenced areas (Zheng et al., 2018; Doré et al., 2022). As climate change alters the defining wind and water circulation patterns of the South China Sea, diminished water exchange with the Pacific Ocean may drive accelerated heating and reductions in nutrient supply to this region (DeCarlo et al., 2017; Tang et al., 2020). Summer heat waves and Fe limitation have in fact been intensifying over the past decades in this region (Wu et al., 2003; Tan et al., 2022; Wen et al., 2022). Mackey et al. (2015) detailed divergent responses of coastal and oceanic Atlantic strains of *Synechococcus* to Fe limitation, but how this may be constrained by warming or influenced by differing Fe regimes remains unclear. This study uses proteomics to explore the connection between temperature and Fe with new isolates from the South China Sea to further our understanding of these interrelated climate stressors.

## 2 Materials and methods

### 2.1 Strains and experimental conditions

Strains were isolated in April 2014 by Q. Zheng. *Synechococcus* sp. YX04-1 was isolated using PRO2 liquid medium from surface waters of the oligotrophic South China Sea (17°N, 112°E), and is a member of clade II within subcluster 5.1A. The present study is the first to report strain YX04-1. *Synechococcus* sp. XM-24 originated in the more dynamic and nutrient-rich surface waters of the coastal Xiamen estuary region of the South China Sea (24°N, 118°E), and is from clade CB5 in subcluster 5.2 (Zheng et al., 2018). Strain XM-24 was isolated in SN medium with reduced salinity (Waterbury et al., 1986; Zheng et al., 2018; Supplementary Figure S1). Genomic sequencing of strains was performed on the Illumina MiSeq (Illumina, San Diego, CA, United States) platform using the MiSeq reagent V2 Kit chemistry and a paired-end 2 × 250 bp cycle run.

Unialgal cultures of each strain were grown in triplicate 1L polycarbonate bottles in temperature-controlled incubators. Cool white fluorescent light was supplied following a 12:12 light:dark cycle under 30 μE m^−2^ s^−1^ irradiance. Cultures were grown in 0.2 μm-filtered, microwave-sterilized Aquil synthetic ocean seawater (Sunda et al., 2005) enriched with 100 μM nitrate, 10 μM phosphate, 25 μM EDTA, a modified Aquil trace metal stock (for final metal concentrations of 1.21 × 10^−7^ M Mn, 7.97 × 10^−8^ M Zn, 1.00 × 10^−7^ M Mo, and 5.03 × 10^−8^ M Co), Aquil vitamins, and 250 nM Fe (for replete cultures only). Fe deplete media was used to dilute Fe limited cultures, and a final concentration of 2 nM Fe was added directly to Fe limited culture bottles on dilution days. Trace metal clean laboratory techniques were followed throughout the experiment to minimize Fe contamination and contaminating Fe was removed from macronutrients (N, P) by passing stocks through an activated Chelex 100 resin column (BioRad Laboratories, Hercules, CA, United States). All nutrients, vitamins, and trace metal stocks were made with microwave-sterilized MilliQ and filtered through a sterile 0.2 μm syringe filter. Nutrients and other stocks were added to the media with sterile pipette tips rinsed in 10% trace metal clean grade HCl followed by sterilized MilliQ. Prior to use, all culture and media bottles were soaked in a 1% Citranox bath overnight, rinsed with MilliQ, soaked in 10% HCl for one week, rinsed again with MilliQ, and microwave-sterilized.

Cultures were diluted semi-continuously every two days with the appropriate media (i.e., Fe replete or Fe deplete, as defined above) to replenish nutrients and sustain steady state exponential growth. Semi-continuous dilutions ensure cells do not enter the early lag or late stationary phases, but instead remain in the mid-exponential growth phase where population growth rates are consistent, and overcrowding or competition for resources do not limit growth. Both strains were maintained under two Fe concentrations, 250 nM and 2 nM, which represented Fe replete and Fe limited treatments, respectively, at two temperatures, 27°C and 30°C, representing an optimum and a supra-optimum growth temperature, respectively. This factorial experimental design allows for an analysis of warming and Fe limitation both individually and concurrently to mechanistically establish their effects on physiological and proteomic responses. For simplicity, the following treatment abbreviations are used throughout the paper: 27R (27°C Fe replete treatment), 30R (30°C Fe replete treatment), 27L (27°C Fe limited treatment), and 30L (30°C Fe limited treatment). After acclimating to the experimental conditions for at least 8 generations, cells were sampled for physiology and protein analysis. Sampling was carried out mid-day, and procedures were consistent between strains.

### 2.2 Physiology sampling procedures

Particulate organic carbon (POC) was measured following previously established methods (Jiang et al., 2018; Yang et al., 2021). Briefly, 30–50 mL of culture was sub-sampled from each experimental triplicate and filtered onto pre-combusted glass microfiber filters (Whatman, Grade GF/F). Filters were dried in an oven at 60°C for 2–3 days before being pelleted and analyzed on a 4,010 Costech Elemental Analyzer calibrated with an acetanilide-based standard curve. POC was used to calculate specific growth rates (μ) using the equation:


$$\mu =\frac{\left(\ln N_{1}-\ln N_{0}\;\right)}{t}$$

Where *N*_1_ and *N*_0_ refer to POC content (μM) of each triplicate during final and initial sampling, respectively, and *t* is time in days between initial and final sampling (2 days). Growth rates (d^−1^) were calculated for each replicate and averaged by treatment.

Intracellular Fe normalized to intracellular phosphorus (P) were used as a proxy for cellular Fe quotas (Fe:P ratios, mmol:mol). Samples for measuring intracellular Fe and P content were digested and assessed via inductively coupled plasma mass spectrometry (ICP-MS, Element 2, Thermo Fisher Scientific) calibrated with a 0.1–300 ppb metal reference standard curve (Hawco et al., 2021; Yang et al., 2021). Briefly, 100 mL of culture was sub-sampled from each experimental triplicate and then filtered on 0.2 μm Supor polyethersulfone filters (Pall Laboratory). Sample filters, along with 3 filter blanks per treatment, were rinsed with 5 mL of 0.2 μm-filtered oxalate reagent for 5 min to remove extracellular Fe (Tovar-Sanchez et al., 2003), rinsed again with trace metal clean natural seawater, and stored at -20°C until analysis. Filters were acid-digested in 5 mL of 50% nitric acid for five days in perfluoroalkoxy vials (Savillex) with the addition of 10 ppb Indium (^115^In) as an internal standard. Dried samples were resolubilized in 200 μL nitric acid and HCl, sealed, heated for 2–3 h, allowed to cool and dry, and then resuspended in 5 mL of 0.1 M distilled nitric acid prior to ICP-MS. Final Fe and P concentrations for each sample, after acid and filter blank intensities were subtracted from sample intensities, were used to calculate Fe:P ratios. All filtration and sampling steps were conducted in a class 100 trace metal clean environment, and all supplies were soaked in 10% HCl for one week and rinsed with Milli-Q prior to use.

### 2.3 Protein extraction and identification

For both strains, 200 mL of each triplicate culture were filtered onto 0.2 μm Supor polyethersulfone filters, and filters were flash frozen in liquid nitrogen and stored until analysis. See Supplementary methods for detailed proteomics analysis steps. Briefly, proteins were extracted using protein extraction buffer (50 mM HEPES pH 8.5, Boston BioProducts) and digested via magnetic beads (SpeedBead Magnetic Carboxylate Modified Particles, GE Healthcare). Tryptic peptides were analyzed via liquid chromatography tandem mass spectrometry (LC/MS/MS) using a Michrom Advance HPLC system with reverse phase chromatography coupled to a Thermo Scientific Q-Exactive Orbitrap mass spectrometer with a Michrom Advance CaptiveSpray source. Scaffold 3 (version 5.1.2, Proteome Software, Inc.) was used to visualize and process the proteomics data, and spectra were converted to normalized total spectral counts (Spectral Counts) (see Supplementary methods).

### 2.4 Functional annotation and data preparation

A functional annotation pipeline entailing DIAMOND (Buchfink et al., 2021), Blast2GO (Conesa et al., 2005; Götz et al., 2008), InterProScan (Jones et al., 2014), UniProt (The UniProt Consortium, 2023), and KofamScan (Kanehisa and Goto, 2000; Aramaki et al., 2020) was carried out to functionally annotate the genomes of each strain (Chille et al., 2021; Yang et al., 2022). Annotations of differentially abundant proteins (DAPs) were manually verified, and in rare cases of discrepancies between KEGG Orthology (KO) and BLAST (from DIAMOND) annotations, the closest BLAST match was used for functional annotation. In the case that multiple proteins corresponded to the same KO annotation, their abundances were summed together so that no duplicate KO annotations remained. Then, proteins which did not have an average abundance of at least 2 (average abundance of the three replicates per treatment) for all treatments were removed to filter out proteins with consistently low abundance that could obscure proteome analyses. The normalized, KO-summed, and filtered protein dataset for each strain was used for all analyses described in the study.

### 2.5 Proteome differential abundance analysis

The Power Law Global Error Model (PLGEM) v1.62.0 R package was used to evaluate DAPs between selected treatments (Pavelka et al., 2004). PLGEM uses the experimental protein data to model signal-to-noise ratios and takes into consideration the typically high amount of random variation in small proteomics datasets (Pavelka et al., 2004, 2008; Walworth et al., 2016; Cohen et al., 2018).

To qualify the proteins which responded to warming alone, we compared each 30°C Fe treatment with its corresponding Fe treatment at 27°C (30R vs. 27R and 30L vs. 27L). To capture DAPs responding to Fe limitation alone, we compared each Fe limited temperature treatment with the Fe replete treatment at the same temperature (27L vs. 27R and 30L vs. 30R). The response of each strain’s proteome to simultaneous warming and Fe limitation was determined through a comparison of the 30L treatment with the 27R treatment (30L vs. 27R).

PLGEM was run in step-by-step mode with default settings. To build the model, a best fit was calculated using one of the treatments within the pairwise comparison. Observed and resampled signal-to-noise ratios were calculated using the selected model fit and used to obtain *p* values and generate a list of DAPs with a false positive rate (FPR) < 0.01. For this study, proteins that additionally had a log2 fold change greater than or equal to 1 (doubling of abundance) or less than or equal to-1 (halving of abundance) were considered significantly differentially abundant (Li et al., 2019). Log2 fold change for each protein was calculated by taking the log_2_(Spectral Count+1) so log values could be calculated for proteins with abundance scores of 0. This method minimally alters the log2 fold change values, but does so consistently across the entire dataset so that trends remain unchanged, and proteins can be compared directly to one another. DAPs were assigned functional categories based on their Kyoto Encyclopedia for Genes and Genomes (KEGG) Pathway and BRITE hierarchy, where similar pathways were merged into the broader functional categories used in the paper.

### 2.6 Statistical analyses and visualizations

All statistical analyses and visualizations were conducted in R v4.0.4. The statistical relationships between growth rates and Fe:P ratios were assessed using a two-way analysis of variance (ANOVA) followed by Tukey HSD post hoc analysis (*p* < 0.05). The same methods were used to detect statistical significance in the differences between mean protein abundances for all box plot visualizations.

Ordinations and multivariate tests were conducted using the vegan v2.6–4 package (Oksanen et al., 2022). Principal component analysis (PCA) was executed as an unconstrained redundancy analysis (RDA) using the ‘rda’ function with log-transformed protein abundance data and using Euclidean distance. The vectors showing direction and degree of the experimental variables, temperature and Fe limitation (-Fe), were calculated using the ‘envfit’ function and then extracting vector scores and scaling them by the ‘ordiArrowMul’ function. An ANOVA–like permutation test was performed by applying the ‘anova.cca’ function to the output from a constrained RDA generated using the ‘rda’ function with 1,000 permutations during one step.

Bar plots, box plots, and ordinations were generated using ggplot v3.4.1, and ggvenn v0.1.9 was used to make Venn diagrams. Heatmaps were generated using pheatmap v1.0.12, and Z-scores for each protein were calculated by dividing the difference between the row-wise mean abundance and the replicate protein abundance by the row-wise standard deviation. Mapping of the isolation sites of *Synechococcus* strains was done using ggplot2 v3.4.2 with all spatial features (ocean, land, and bathymetry layers) downloaded from Natural Earth.^1^

## 3 Results

### 3.1 Physiological responses of each strain to temperature and Fe treatments

**Figure 1:**
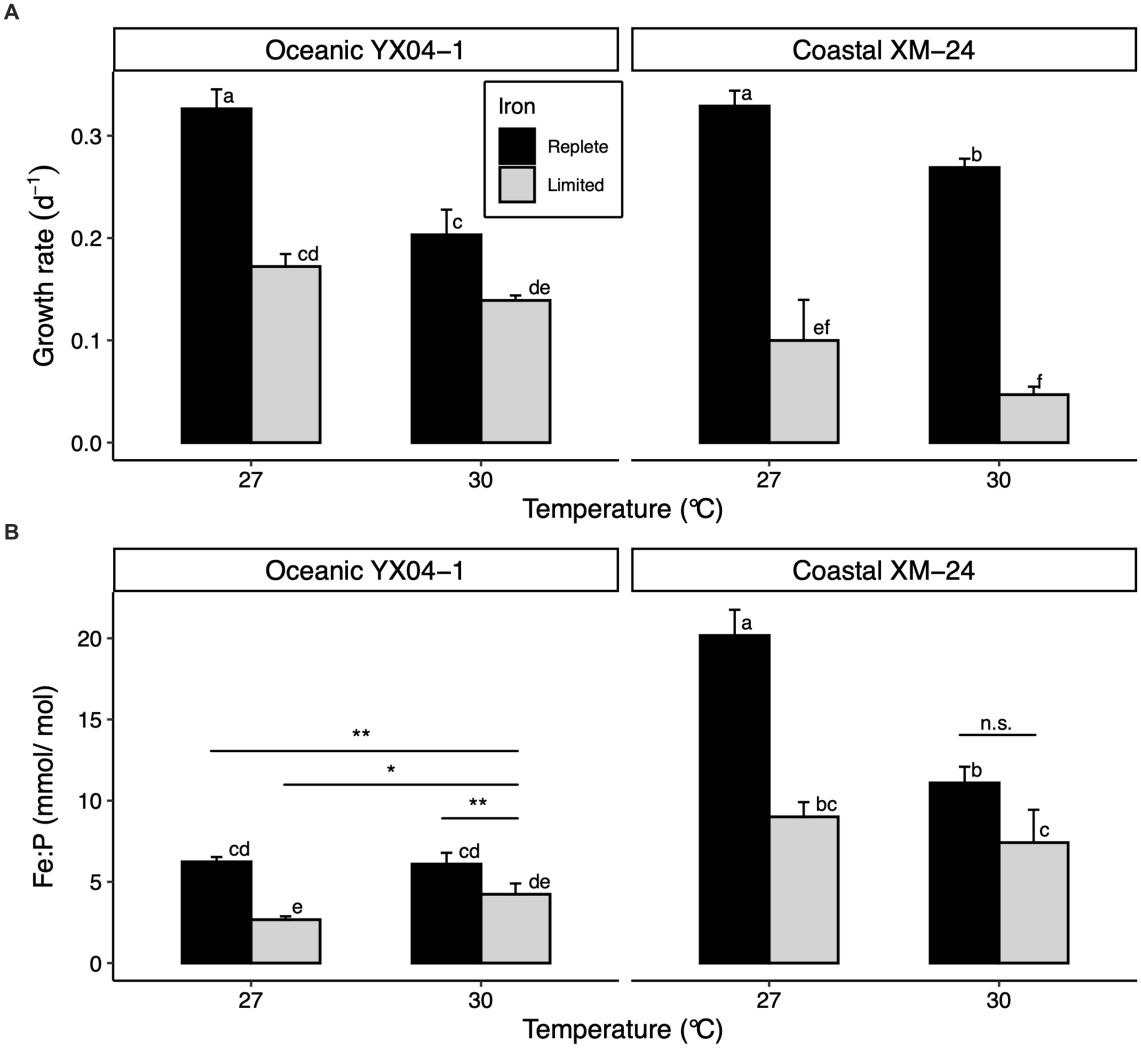
Physiology of oceanic *Synechococcus* strain YX04-1 and coastal *Synechococcus* strain XM-24 grown under two temperatures and two iron concentrations. **(A)** Specific growth rates and **(B)** intracellular iron: phosphorus (Fe:P) ratios. Letters above bars denote differences between treatment means across both strains, where non-overlapping letters signify statistically significantly different means (*p* < 0.05) within each assay. Asterisks (*) represent differences between treatment means within each strain as opposed to across strains, with the significance thresholds: * = *p* < 0.05, ** = *p* < 0.01, and n.s. = *p* > 0.05. For simplicity, within-strain significance is only shown where it differs from between-strain significance. Statistical significance was calculated using two-way ANOVA and Tukey HSD *post hoc* testing. Error bars represent standard deviation of three replicates in each treatment, except Fe:P ratios for YX04-1’s 27°C Fe replete treatment, which is represented by two replicates.

Acclimating to low Fe predictably slowed growth of both strains at both temperatures, though coastal XM-24 experienced a greater decline in growth in response to Fe limitation at each temperature compared with oceanic YX04-1 (Figure 1A). Regardless of Fe condition, cells acclimated to 27°C grew faster than those grown at 30°C for each strain. Growth rates between strains were comparable at 27°C under replete Fe (27R). However, coastal XM-24 grew faster than oceanic YX04-1 when warmed to 30°C under replete Fe (30R), while YX04-1 outgrew XM-24 at 30°C under limiting Fe (30L). Under the 30L treatment, which represents the combined effects of warming and Fe limitation when compared to the 27R treatment, growth of the oceanic strain showed a 57% reduction, compared with a larger 86% decline in the growth of the coastal strain.

Overall, Fe:P ratios (Fe quotas) were significantly lower for oceanic YX04-1 than for coastal XM-24 at each temperature (Figure 1B). As expected, Fe limitation significantly decreased each strain’s Fe quotas at both temperatures, except for XM-24 30°C treatments. Warming affected each strain’s Fe quotas differently; while warming to 30°C reduced coastal XM-24’s Fe quotas across both Fe conditions, it did not affect oceanic YX04-1’s Fe quotas under Fe replete conditions and caused them to increase under Fe limitation.

### 3.2 Overall trends in the global proteomes

Proteomics analyses identified 781 proteins for oceanic YX04-1, covering 31% of its 2,557 predicted coding regions, and 1,010 proteins for coastal XM-24, representing 41% of its 2,477 predicted coding regions, across 12 samples for each strain. After annotating and trimming steps, the oceanic and coastal proteomes consisted of 617 and 677 unique proteins, respectively (Supplementary Table S1). Under Fe limitation, the oceanic strain demonstrated major reductions in the size of the proteome. Acclimation to the 27°C Fe limited treatment (27L) diminished its measured proteome to 64% of its size under the optimum growth conditions (27R), and warming intensified this response, leading to a further reduction in the 30L treatment to 51% of its original proteome size (Supplementary Figure S2). The coastal strain retained a larger proportion of its proteome under Fe limitation compared with the oceanic strain, reducing its proteome to 82 and 75% of its original size when grown in the 27L and 30L treatments, respectively (Supplementary Figure S2).

Principal components analysis (PCA) and an ANOVA-like permutation test suggest that Fe limitation has the strongest influence on protein abundances (proteome variation) for both strains (*p* = 0.001), while temperature alone is less likely to influence changes in proteome variation (*p* = 0.05) for both strains (Supplementary Figure S3). The PCA further indicates that Fe has a stronger effect on the whole proteome variation of oceanic YX04-1 than coastal XM-24 at each temperature, contrasting with the greater physiological response of XM-24 to Fe limitation.

### 3.3 General proteomic responses to warming, Fe limitation, and their concurrence

**Figure 2:**
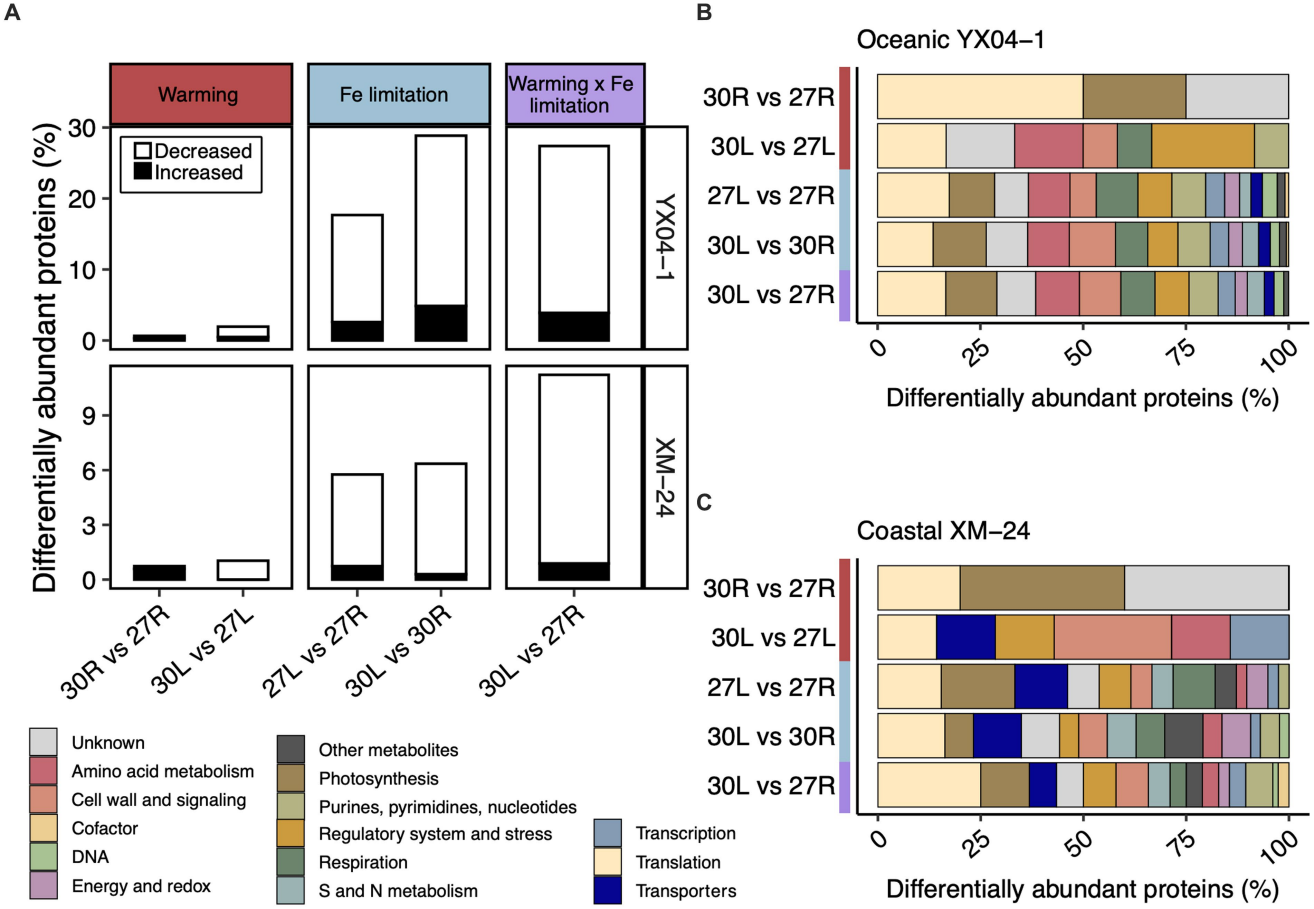
Summary of the proteomic responses from each pairwise comparison. **(A)** The proportion of differentially abundant proteins (DAPs) in each pairwise comparison representing the effect of warming, Fe limitation, and the concurrence of warming and Fe limitation for oceanic strain YX04-1 (top) and coastal strain XM-24 (bottom). Functional categories of DAPs in each pairwise comparison for **(B)** oceanic strain YX04-1 and **(C)** coastal strain XM-24. Red, blue, and purple represent the warming, Fe limitation, and warming x Fe limitation (interaction) scenarios in all plots, while functional categories are represented by colors defined in the figure key. The relative abundance of proteins for **(A)** was calculated by dividing the number of DAPs in each comparison by the total number of proteins in the dataset. Note difference in the y axis scales between strains. The relative abundance of functional categories for **(B,C)** was calculated by dividing the number of DAPs in each functional category within a given comparison by the total number of DAPs in that comparison.

We used the Power Law Global Error Model (PLGEM) to compare the DAPs from pairwise comparisons representing warming alone (30R vs. 27R and 30L vs. 27L), Fe limitation alone (27L vs. 27R and 30L vs. 30R), and simultaneous warming and Fe limitation (30L vs. 27R) (Figure 2; Supplementary Table S2). In total, 207 distinct proteins, representing 33.5% of its detected proteome, made up oceanic YX04-1’s response to all five pairwise comparisons. 96 distinct proteins, or 14.2% of its detected proteome, composed coastal XM-24’s total response. In most pairwise comparisons, both strains significantly decreased a large proportion of their DAPs, especially under Fe limitation or its concurrence with warming (Figure 2A). Pairwise comparisons between 30°C and 27°C under each Fe condition indicated that warming alone had a minimal effect on protein abundances of both strains, as less than 1% of their proteomes responded (Figure 2A, left column). Fe limitation at each temperature induced a greater proteomic response than warming alone, underscoring the greater influence of Fe limitation on the proteomes of both strains (Figure 2A, middle column). A greater proportion of the oceanic strain’s proteome responded to Fe limitation with 18 and 29% responding at the optimum and supra-optimum temperatures, respectively, compared with 6% of the coastal strain’s proteome that responded to both Fe limitation scenarios. The proportion of oceanic YX04-1’s proteome that responded to the interaction scenario was similar to its response under Fe limitation at the supra-optimum temperature, whereas the proportion of coastal XM-24’s DAPs in the interaction scenario increased to 11% of its proteome (Figure 2A, right column).

### 3.4 Prominent cellular functions in response to each scenario

Categorizing the DAPs in each pairwise comparison suggests that DAPs in the different warming and Fe limitation scenarios involve similar cellular functions (Figures 2B,C). When functional categories of proteins from all pairwise comparisons were summed together, translation and photosynthesis were the two most responsive overall categories in both strains. As warming alone prompted very few DAPs, it was impractical to include them for further consideration in our analysis.

In each of the three pairwise comparisons making up the Fe limitation and interaction scenarios, translation and photosynthesis were the two most relatively abundant functional categories for oceanic YX04-1 (Figure 2B). For coastal XM-24, translation was most relatively abundant in the 30L vs. 30R and 30L vs. 27R comparisons, while photosynthesis had the greatest relative abundance in this strain’s 27L vs. 27R comparison (Figure 2C). Translation and transporters were the second most relatively abundant categories in coastal XM-24’s Fe limitation responses at 27°C and 30°C, respectively, while photosynthesis had the second greatest relative abundance in its interaction scenario. In addition to these central cellular functions, less prominent categories including sulfur (S) and nitrogen (N) metabolism, and regulatory system and stress were also important in each strain’s Fe limitation and interaction scenarios (Figures 2B,C).

### 3.5 Proteins responding to Fe limitation and its concurrence with warming

**Figure 3:**
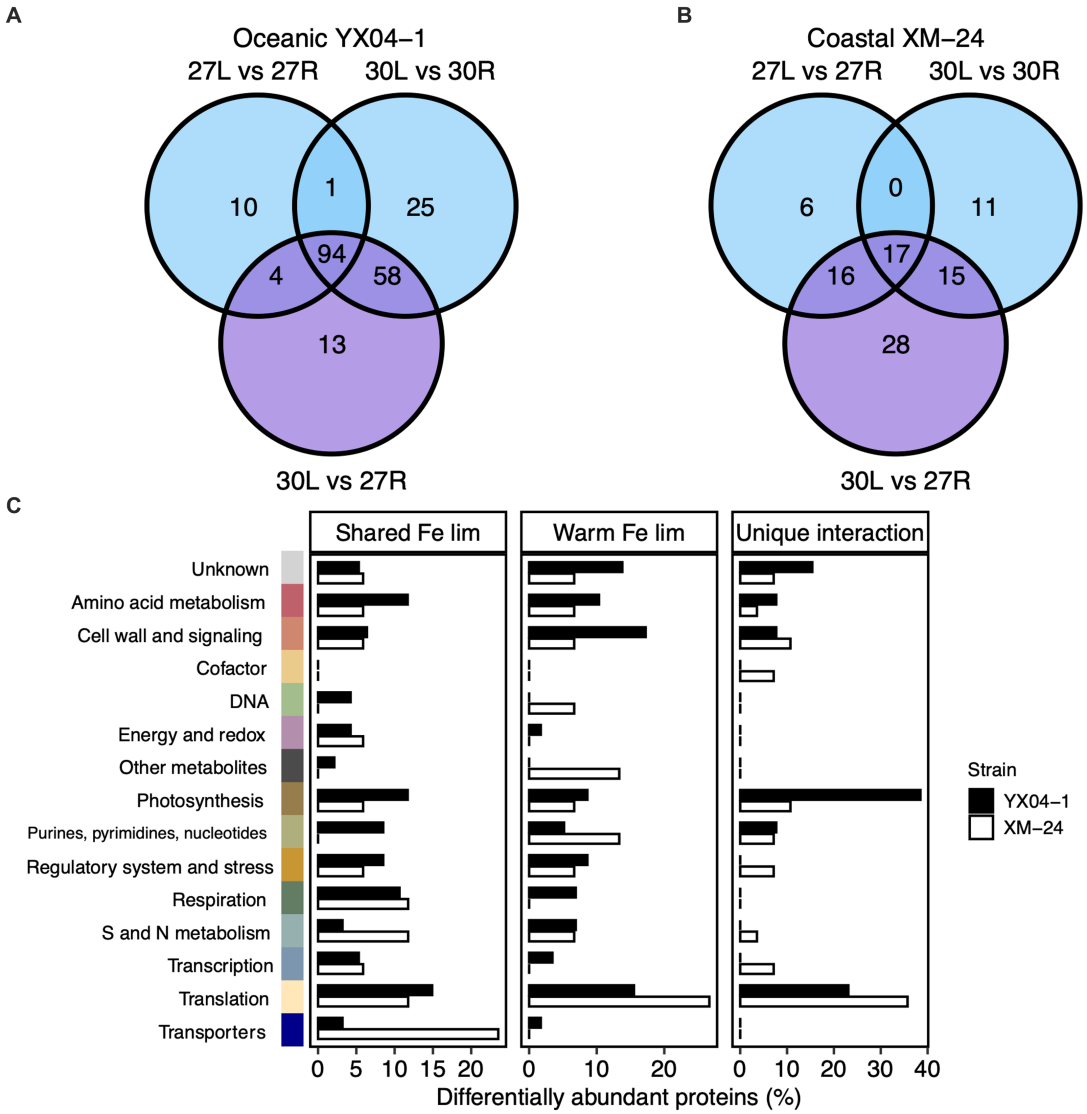
Comparison of differentially abundant proteins in the Fe limitation and interaction scenarios. Venn diagrams of differentially abundant proteins (DAPs) in each of the Fe limitation (blue; 27 L vs. 27R and 30 L vs. 30R) and interaction (purple; 30 L vs. 27R) scenarios for **(A)** oceanic strain YX04-1 and **(B)** coastal strain XM-24. **(C)** Functional categories of proteins within the shared Fe limitation, warm Fe limitation, and unique interaction groups for oceanic YX04-1 (black bars) and coastal XM-24 (white bars). Note differences in x axis scales. DAPs in each functional category are represented by bars for each strain as their relative abundance within each of the three groups. Colors representing functional categories correspond with previous figure. DAPs shared between all three of the Fe limitation and interaction pairwise comparisons are considered in the shared Fe limitation (“lim”) group, DAPs shared only between the 30 L vs. 30R and the 30 L vs. 27R comparisons make up the warm Fe lim group, and DAPs only in the 30 L vs. 27R comparison are within the unique interaction group.

To mechanistically assess the effect of thermal stress on acclimation to Fe limitation within each strain, we compared the DAPs shared between all three of the Fe limitation and interaction scenarios, or the “shared Fe limitation response,” with the proteins that were shared only between the 30L vs. 30R and the 30L vs. 27R comparisons, or the “warm Fe limitation response.” We additionally considered the proteins which responded exclusively to the interaction scenario, or the “unique interaction response” of each strain (Figure 3; Supplementary Table S3).

Comparing proteins from the Fe limitation and interaction scenarios revealed high overlap between these scenarios for oceanic YX04-1 but not for coastal XM-24. 94 proteins, or 46% of the oceanic strain’s DAPs made up its shared Fe limitation response, and an additional 28%, or 58 proteins, were part of this strain’s warm Fe limitation response. Meanwhile, only 13 proteins, representing 6% of its total response, were unique to its interaction scenario (Figure 3A). On the other hand, a lower proportion of proteins composed the coastal strain’s shared Fe limitation response, with 17 proteins, (18%), and its warm Fe limitation response, consisting of 15 proteins, or 16% of its total protein response. A larger proportion was unique to this strain’s interaction response, represented by 28 proteins, or 30% of its DAPs (Figure 3B).

**Figure 4:**
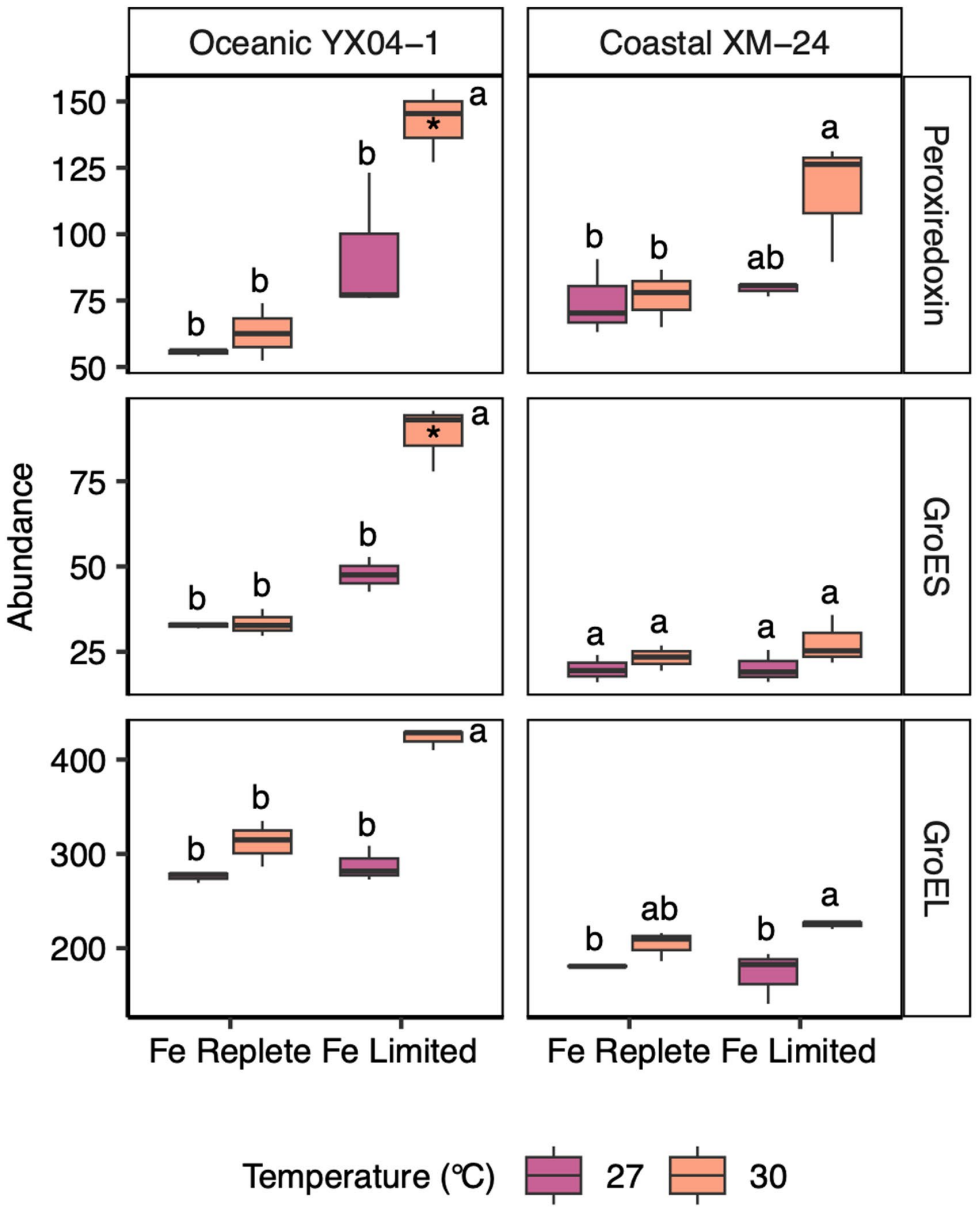
Abundances of oxidative stress and chaperonin proteins for oceanic YX04-1 (left) and coastal XM-24 (right). A peroxiredoxin and two chaperonins, GroES and GroEL, are listed on the righthand side of each row. Box plots represent treatment means of 3 replicates, and unique letters denote statistically significant means (within strain) for each protein, based on a two-way ANOVA and Tukey *post hoc* testing (*p* < 0.05). (*) Note that in the study, only the peroxiredoxin and GroES of YX04-1 were considered significantly differentially abundant in the 30 L treatment based on the more stringent PLGEM and Log2 fold change thresholds.

**Figure 5:**
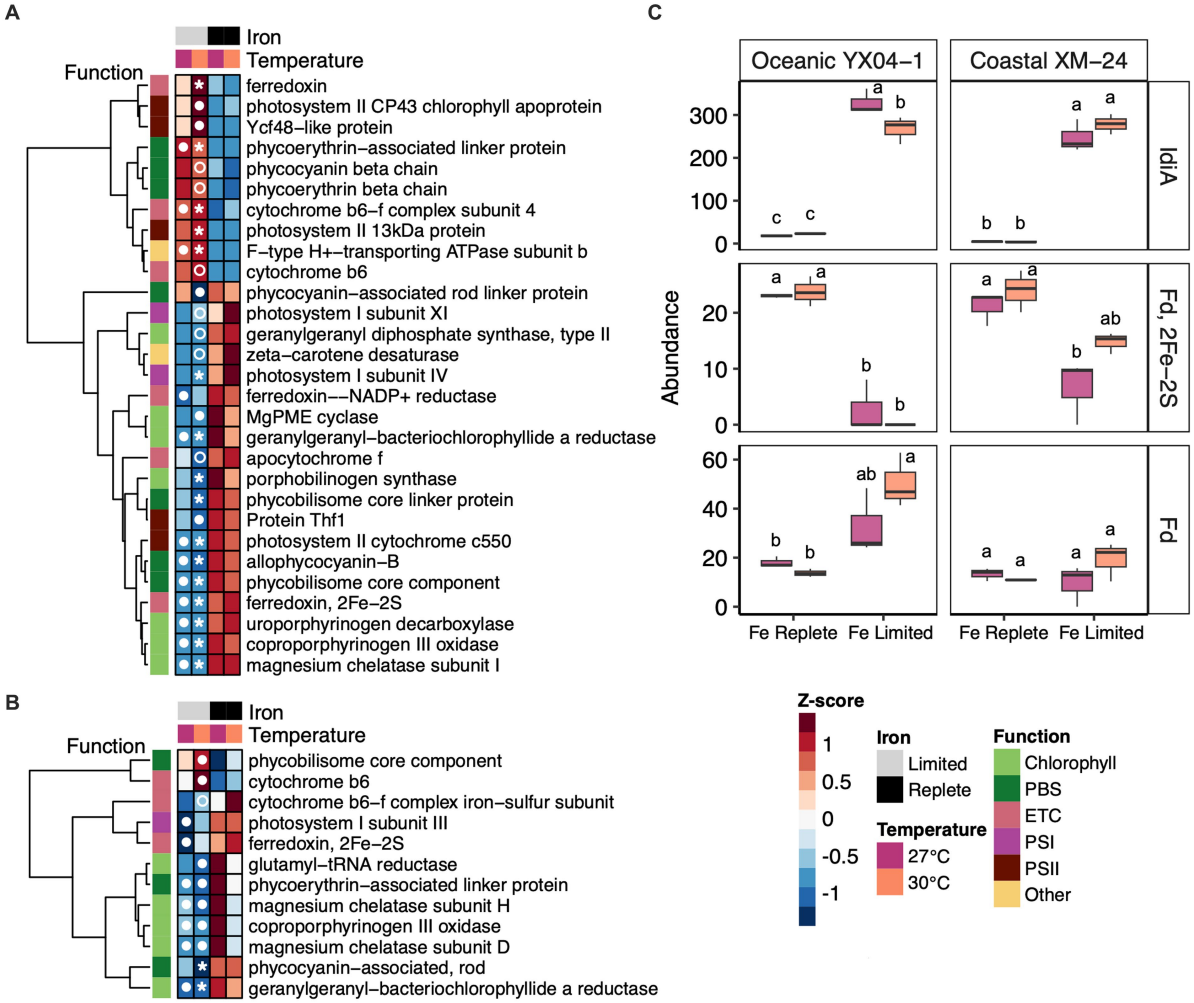
Abundance trends of selected photosynthesis and Fe response proteins. Heat maps showing abundances of differentially abundant photosynthesis proteins in all of the Fe limitation and interaction scenarios for **(A)** oceanic strain YX04-1 **(B)** coastal strain XM-24. **(C)** Abundances of IdiA/FutA and two paralogs of ferredoxin (Fd) for oceanic strain YX04-1 (left) and coastal strain XM-24 (right). Box plots in **(C)** represent protein abundances of 3 replicates for each treatment, and letters showing statistical significance were calculated using two-way ANOVA and Tukey HSD *post hoc* testing where unique letters represent significantly different treatment means (*p* < 0.05) within each strain. Protein abundances for heat maps were scaled using row-wise Z-score normalization. Symbols in heat map cells represent in which pairwise comparison each protein is significant, as follows: 27 L vs. 27R (closed circle in the 27 L cell), 30 L vs. 27R (closed circle in the 30 L cell), 30 L vs. 30R (open circle in the 30 L cell), and both 30 L vs. 30R and 30 L vs. 27R (star in the 30 L cell). The photosynthetic function of each protein in heat maps is indicated by column annotations as defined in the figure key.

In its shared Fe limitation response, oceanic YX04-1 primarily increased Fe transport proteins, namely an IdiA/ FutA homologue and two putative Fe uptake porins, and a small number of photosynthesis proteins including subunit IV of the cytochrome b6f complex. Many more proteins declined in abundance, which were mainly related to translation and amino acid metabolism, photosynthesis and chlorophyll synthesis including a 2Fe-2S ferredoxin paralog, and S and N assimilation including ferredoxin-dependent nitrite and sulfite reductases (Figure 3C). The oceanic strain’s warm Fe limitation response was defined by increases in some proteins including ribosomal proteins, stress response proteins including the antioxidant peroxiredoxin and chaperonin GroES (Figure 4), a component of photosystem II (PSII; Psb28), and a different ferredoxin paralog. This response also consisted of further decreases in fatty acid synthesis proteins, other photosynthesis proteins including a photosystem I (PSI) component (PsaE), and urea metabolism, namely a urease subunit alpha and urea transport system ATP-binding protein. In its comparatively small set of proteins in the unique interaction response, oceanic YX04-1 increased abundance of other PSII components, including CP43 and PSII assembly factor Ycf48, while also further decreasing the abundance of some other photosynthesis and translation related proteins (Figures 3C, 5A). All combined, Fe limitation occurring at 30°C resulted in significant changes to photosystem components, shifts in ferredoxin abundances, decreases in urea metabolism, and an enhanced antioxidant defense compared with Fe limitation at 27°C.

Coastal XM-24’s shared Fe limitation response was smaller than oceanic YX04-1’s and consisted of increases in Fe uptake proteins and decreases in proteins primarily involved in S and N metabolism including ferredoxin-dependent nitrate reductase, translation, cell wall synthesis, and respiration (Figure 3C). XM-24 did not increase abundance of any proteins in its warm Fe limitation response, instead further decreasing translation proteins, metabolites including heme oxygenase, and a urease subunit alpha. Unlike the oceanic strain, its unique interaction response was composed of the largest proportion of proteins out of all groups in this strain. Coastal XM-24’s unique interaction response revealed that the concurrence of warming and Fe limitation led to increases in photosynthesis related proteins including cytochrome b6, as well as a branched-chain amino acid aminotransferase, and decreases in cell wall and signaling, translation proteins, and S proteins including a sulfite-reductase and Fe-S cluster assembly protein (Figures 3C, 5B).

### 3.6 Recurring Fe response proteins and their relationship with temperature

Regardless of whether Fe limitation was induced at a single temperature (27L vs. 27R and 30L vs. 30R) or co-occurred with warming (30L vs. 27R), Fe limitation prompted both strains to increase abundances of Fe transport proteins. Iron deficiency-induced protein A (FutA/ IdiA) homologues (Figure 5C, top panel) and putative Fe uptake porins (Supplementary Table S2) were among the most highly increased and most abundant proteins in each strain’s proteomic response to these scenarios.

Other Fe-related proteins also played a role in each strain’s responses to Fe limitation, with some influenced interactively by the concurrence of warming with Fe limitation. Two ferredoxin paralogs showed significant changes in abundance under Fe limitation, though temperature and Fe limitation influenced their abundances in different ways. Both strains decreased abundance of one ferredoxin paralog, identified as a 2Fe-2S ferredoxin, under Fe limitation, though its decline was not significant in coastal XM-24’s 30L treatment (Figure 5C, middle panel). Another paralog of ferredoxin, annotated as Ferredoxin, responded oppositely to Fe limitation in oceanic YX04-1 compared with the 2Fe-2S ferredoxin; low Fe drove significant increases in its abundance in the 30L treatment compared with both replete temperature treatments (Figure 5C, bottom panel). This paralog was also found in coastal XM-24 but did not respond significantly to Fe or temperature.

Aside from ferredoxin, components of another electron carrier, the cytochrome b6f complex, responded to changes in Fe and temperature in both strains (Supplementary Figure S4). Subunit IV of the cytochrome b6f complex increased in all three of the Fe limitation and interaction scenarios for oceanic YX04-1, and cytochrome b6 also increased in its 30L vs. 30R comparison. Similarly, cytochrome b6 increased in response to these scenarios for coastal XM-24, but only significantly in its interaction scenario. Thus, in both strains, these components of their cytochrome b6f complex increased under Fe limitation or its concurrence with warming. The Fe-S and cytochrome f subunits of the cytochrome b6f complex were also differentially abundant in these treatments (see Discussion).

S and N assimilation proteins were also responsive in the Fe limitation and interaction scenarios (Supplementary Figure S5; Supplementary Table S2). In the oceanic strain, three ferredoxin-dependent S and N assimilation proteins (ferredoxin-dependent glutamate synthase, nitrite reductase, and sulfite reductase) decreased in all three comparisons within the Fe limitation and interaction scenarios; Fe limitation reduced their abundance regardless of temperature. Ferredoxin-dependent nitrate reductase also declined significantly in these scenarios for coastal XM-24, but this protein was not detected in the proteome of oceanic YX04-1. Warming in addition to Fe limitation led to synergistic declines in abundance of a urease subunit alpha in both strains’ 30L treatment relative to both the 27R and 30R treatments.

## 4 Discussion

This study investigated how the diversity of marine *Synechococcus* isolated from the South China Sea controls their capacity to acclimate to shifts in two linked climate stressors, warming and Fe limitation. The strains selected in this study originated from contiguous coastal and oceanic regions defined by distinct temperature and Fe regimes. They also belong to different clades and subclusters of *Synechococcus*, highlighting their genetic divergence. We observed significant physiological differences in response to warming and Fe limitation between strains that were likely driven by ecotype-level diversity, and our proteomic analysis further explored how shifts in metabolic strategies of each strain led to the observed physiological outcomes.

### 4.1 Opposite interactive effects of temperature and Fe limitation between strains

Warming decreased growth rates of the oceanic and coastal strains under both Fe conditions, although the magnitude of these trends was strain-specific and Fe-dependent. Under replete Fe, coastal XM-24 growth rates declined less in response to warming than those of oceanic YX04-1, suggesting it may be well adapted to the warmer waters of its coastal environment in the South China Sea. As climate change continues to progress, stressful warm temperatures are expected to become even more frequent and severe in this region (Yao et al., 2020). Despite the observed physiological changes, both environmentally realistic warming scenarios prompted only minor proteomic responses. These results align with the multivariate statistical analysis and demonstrate the limited impact of warming across this temperature range on the overall proteome variation in both strains.

These growth trends with warming were reversed under Fe limitation; oceanic YX04-1’s growth was less negatively affected than coastal XM-24’s by warming under Fe limitation, compared with under Fe replete conditions. The effect of Fe limitation on the oceanic strain’s response to warming supports findings from a previous study which found Fe limitation to mitigate the deleterious effect of warming on growth rates of the N-fixing cyanobacterium *Trichodesmium*. Their study additionally reported an upward shift in the strain’s thermal optimum under Fe limitation, which was not observed in our strains (Jiang et al., 2018). Others have found similar interactive effects between temperature and Fe limitation on cyanobacteria and phytoplankton physiology (Jabre and Bertrand, 2020; Yang et al., 2021), while still others have observed antagonistic interactive effects between warming and Fe limitation (Sunda and Huntsman, 2011; Andrew et al., 2019).

The nearshore region where XM-24 was isolated experiences higher Fe and nutrient inputs than the offshore oligotrophic habitat of YX04-1, as is common in coastal regions (Sunda and Huntsman, 1995; Miao et al., 2006; Twining et al., 2021). Oceanic YX04-1’s superior growth under Fe limitation at both temperatures may be attributed to traits that have been shaped by adaptation to the lower Fe environment where it was originally isolated. Physiologically, this is demonstrated by their Fe:P ratios, where the oceanic strain has significantly lower Fe quotas across all treatments compared with the coastal strain.

Oceanic YX04-1’s previous adaptations to a low Fe habitat have likely resulted in a more flexible proteome, allowing it to quickly alter metabolic needs in response to low Fe. Its ability to reduce the overall size of its proteome, and many of its Fe-requiring proteins, may explain this strain’s superior growth under low Fe compared with coastal XM-24. A larger measured response, indicated by a greater number of differentially abundant proteins, indicates that cells are experiencing higher levels of stress in the given growth conditions. At the same time, it can demonstrate that they possess a robust and well-developed system to effectively respond to stressors (Mackey et al., 2015; Walworth et al., 2016). Supporting this, the proportion of oceanic YX04-1’s DAPs that responded to the Fe limitation and interaction scenarios was more than double that of coastal XM-24. The oceanic strain’s larger proteomic response may contribute to the overall growth success of this strain under Fe limitation at both temperatures.

The findings here differ from those of Mackey et al. (2015), who found a coastal strain of *Synechococcus*, WH8020 from the New England shelf, to outperform oceanic strain WH8102 from the southern Sargasso Sea under multiple levels of Fe limitation (Mackey et al., 2015). In contrast with the low offshore Fe levels in the South China Sea basin, the higher, stable levels of Fe in the southern Sargasso Sea resulting from consistent dust deposition likely caused oceanic WH8102 to lose Fe response genes compared with coastal WH8020 (Mackey et al., 2015). Further, different clade designations between strains YX04-1 (clade II) and WH8102 (clade III) highlight their phylogenetic divergence. These differences between studies underscore the relevance of considering the influence of Fe ecotypes on *Synechococcus* responses to stressors.

The greater proteomic responses of both strains to Fe limitation compared with warming supports previous knowledge that marine cyanobacteria have evolved specific and tightly-regulated responses to nutrient limitation (Held et al., 2019; Walworth et al., 2022). Ferric uptake regulator (Fur) directs the expression of multiple Fe response genes, enabling cells to quickly respond to changing Fe conditions (Hantke, 1981; Kaushik et al., 2016). Both strains possess three Fur family genes, though they were not detected in the proteomes. On the other hand, *Synechococcus* may lack a specific sensor and response system for temperature, leading to a warming response that is based more loosely on the direct effect of warming on specific proteins, enzyme activities, and degradation rates. Additionally, these two kinds of responses may work together under combined stressors; as oceanic YX04-1’s growth was less negatively impacted by warming when grown under Fe limitation, it is possible that its Fe response aided its acclimation to warming.

### 4.2 Oceanic YX04-1 synergistically modifies photosynthesis under warm, Fe limited conditions

Reductions in Fe-rich PSI, cytochromes, phycobilisomes (PBS), and chlorophyll biosynthesis proteins are characteristic strategies of cyanobacteria to regulate photosynthetic Fe demand in response to Fe deficiency (Fraser et al., 2013; Snow et al., 2015; Sunda and Huntsman, 2015; Blanco-Ameijeiras et al., 2017). In our study, photosynthesis emerged as the second most differentially abundant functional category in each strain, which included proteins involved in chlorophyll biosynthesis, PBS, photosystems, and the photosynthetic electron transport chain (PETC). However, differences in the way they responded to Fe limitation across temperatures suggest varying strategies for photosynthetic acclimation of each strain. For oceanic YX04-1, a greater fraction of its photosynthetic proteins responded to Fe/ temperature co-stress, as evidenced by its 30L comparisons (30L vs. 30R and 30L vs. 27R). Conversely, coastal XM-24’s photosynthetic proteins were most responsive to Fe limitation at the thermal optimum (27L vs. 27R).

Deleterious warming further damages photosynthetic efficiency by weakening thylakoid membrane stability and increasing rates of protein denaturation (Falk et al., 1996; Hutchins and Fu, 2017). Changes to photosynthetic protein arrangement due to Fe or heat stress can result in reduced photosynthetic efficiency and proliferation of harmful ROS, necessitating acclimation strategies to not only reduce Fe demand but also protect protein components from oxidative damage (Bailey et al., 2008; van Creveld et al., 2016). The heightened response of the oceanic strain’s photosynthetic proteins to warm, Fe limited conditions was primarily driven by reorganization of its photosystems, suggesting a synergistic effect of temperature and Fe limitation on the photosynthetic apparatus.

Under ideal conditions, cyanobacteria maintain low PSII:PSI ratios, which helps prevent overreduction of the PETC by ensuring there are sufficient PSI complexes to accept electrons from PSII during linear electron flow (Bailey et al., 2008). However, maintenance of a low ratio is costly, with PSI requiring 12 Fe atoms compared with the 2–3 involved in PSII. Therefore, some cyanobacteria have been shown to preferentially decrease Fe-rich PSI in response to Fe limitation, thus increasing their PSII:PSI ratios (Raven et al., 1999; Snow et al., 2015). Oceanic YX04-1 reorganized its core photosystem proteins via an increase in its PSII:PSI ratio in the Fe limited treatments (Supplementary Figure S6). However, significant differences in photosystem protein abundances primarily occurred only in the oceanic strain’s warm Fe limitation comparisons. Fe limitation at 30°C drove significant reductions in PsaE (PSI subunit IV) abundance in both warm Fe comparisons, and PsaL (PSI subunit XI) additionally decreased in abundance in the 30L vs. 30R comparison. Fe limitation at 30°C also prompted increases in abundances of PSII proteins; PSII reaction center protein Psb28 increased in the warm Fe limitation response, and PsbC (CP43) and PSII assembly protein Ycf48 increased in the interaction scenario. In contrast, Fe limitation did not lead to an increase in the core PSII:PSI ratio of coastal XM-24 (Supplementary Figure S6). XM-24 significantly decreased abundance of one PSI protein, PsaF (PSI subunit III), only in the 27L treatment, while no photosystem components significantly responded to either of its 30L treatment comparisons.

Varying levels of photosystem subunits in response to Fe limitation have also been observed in other phytoplankton. Abundances of PSI proteins decreased while PSII protein abundances either increased or remained the same in Fe limited cultures of *Trichodesmium* (Walworth et al., 2016). Moreno et al. (2020) also observed increased abundances of specific photosystem subunits despite an overall downregulation of photosynthesis in response to Fe and light limitation in a polar diatom species. Different photosynthetic components may play unique roles in the oceanic strain’s photosynthetic acclimation to Fe limitation, especially under warming. The shifts in oceanic YX04-1’s photosynthetic proteins under concurrent stressors indicate a synergistic effect that drove an increased need to reorganize photosynthetic components to cope with enhanced photodamage that can result due to both Fe and heat stress.

Ycf48, which assembles and repairs PSII (Komenda et al., 2008), significantly increased in oceanic YX04-1’s interaction scenario, which could be an indication of increased rates of photodamage brought on by the combination of Fe and heat stress. Thus, while warming alone minimally affected photosynthesis, it interacted with the deleterious effects of Fe limitation to increase rates of photodamage. On the other hand, coastal XM-24’s general lack of significant reorganization of its photosystems implies a relatively higher photosynthetic Fe demand for this strain. Previous studies provide support for the reduced ability of coastal phytoplankton to effectively moderate their photosynthetic Fe requirements (Strzepek and Harrison, 2004; Sunda and Huntsman, 2015).

The cytochrome b6f complex is an essential component of linear photosynthetic electron transport, but has a high Fe demand, and its abundance is often reduced when Fe is limiting (Fraser et al., 2013; Blanco-Ameijeiras et al., 2017). While coastal XM-24 significantly decreased abundance of the Fe-S subunit of the cytochrome b6f complex and oceanic YX04-1 decreased abundance of cytochrome f in each of their 30L vs. 30R scenarios, other components of the cytochrome b6f complex were measured in higher abundances in both strains. Namely, subunit IV increased significantly in oceanic YX04-1’s shared Fe response, and abundance of cytochrome b6 also increased significantly in its 30L vs. 30R comparison. Cytochrome b6 also increased in abundance in coastal XM-24’s interaction scenario. Similarly, increased expression of the cytochrome b6f complex subunit IV gene (*petD*) has been observed in Fe limited *Synechococcus* (Singh et al., 2003). It is possible *Synechococcus* utilize specific components of the cytochrome b6f complex to oversee the redox state of their PETC in order to mitigate oxidative damage caused by the combined effects of heat stress and Fe limitation on electron flow, as has been shown in plants and green algae (Wollman and Lemaire, 1988; Dumas et al., 2017) and hypothesized for cyanobacteria (Mao et al., 2002; Huang et al., 2003). IsiA, a chlorophyll binding protein that associates with PSI to reduce photodamage under Fe limitation (Bibby et al., 2001), was not found in either strain’s genome (see Supplementary Note).

Overall, the comparatively limited response of the coastal strain’s photosynthetic proteins demonstrates a reduced capacity to acclimate to Fe limitation relative to oceanic YX04-1. This deficiency was particularly evident when Fe limitation and warming were combined. In contrast, the oceanic strain’s warming-driven adjustment of photosynthetic proteins under Fe limitation to conserve Fe, repair photodamaged proteins, and reduce generation of ROS likely led to the observed mitigation of warming effects on oceanic YX04-1’s Fe limited growth.

### 4.3 Oceanic YX04-1 enacts a more robust stress response

We observed increases in abundance of antioxidant and stress proteins under the two climate stressors, especially in response to their co-occurrence. In oceanic YX04-1, a peroxiredoxin increased significantly in the 30L treatment. Peroxiredoxin has been reported to defend PSII against oxidative stress under low Fe conditions (Moreno et al., 2020), and here its abundance was even more impacted by the combination of warming and Fe limitation. The abundance of protein chaperone GroES also increased significantly in the oceanic strain’s 30L treatment compared with both Fe replete treatments. GroES works in concert with GroEL to manage misfolded proteins under stressful conditions (Potnis et al., 2016); we did observe increases, though not significant, in YX04-1’s GroEL in the 30L treatment.

The rise in abundance of GroES in oceanic YX04-1 suggests increased protein denaturation under the combination of heat and Fe stress. This is likely connected to an amplification of oxidative stress in the 30L treatment, which is also indicated by increases in peroxiredoxin and shifts in photosynthetic proteins in oceanic YX04-1. The highest abundance of peroxiredoxin and GroES occurred under simultaneous warming and Fe limitation, signaling that warming interacted with Fe limitation to drive the highest levels of oxidative stress in this strain. Heat stress can hinder electron transport and increase oxidative damage (Falk et al., 1996). As a result, cyanobacteria utilize their PBS in state transitions to minimize overreduction of the PETC and reduce the generation of ROS in response to warming (Mackey et al., 2013), but Fe limited conditions such as in our study likely inhibit this acclimation mechanism due to Fe-driven reductions in PBS synthesis.

On the other hand, peroxiredoxin increased slightly but not significantly in coastal XM-24’s 30L treatment. In fact, while multiple stress response proteins were a part of the oceanic strain’s response to Fe limitation, none of these proteins played a role in the coastal strain’s response to any of the experimental scenarios. Ferritin, an Fe storage protein, may normally offer an advantage to coastal XM-24 in its ability to reduce oxidative stress by safely harboring Fe under replete conditions, and directing it to specific processes in the cell under short-term fluctuations in Fe supply (Ahlgren et al., 2020; Gilbert et al., 2022). *Synechocystis* mutants devoid of ferritin grown under Fe limitation exhibited a greater antioxidant response compared with wild type cells (Shcolnick et al., 2009), and ferritin has also been found to be involved in the reduction of oxidative stress in higher plants (Ravet et al., 2009). In XM-24’s dynamic coastal environment, it may be advantageous to rely on ferritin to buffer short-term reductions in Fe supply instead of maintaining the ability to enact a strong oxidative stress response such as in the oceanic strain. However, over the longer period of exposure in this study, the coastal strain’s less robust Fe stress response may have contributed to its reductions in growth rates compared with oceanic YX04-1. Accordingly, coastal XM-24 possesses genes for both a bacterial non-heme ferritin and a bacterioferritin, while no forms of ferritin were found in the genome of YX04-1, consistent with other studies of open ocean *Synechococcus* (Mackey et al., 2015). However, only coastal XM-24’s bacterial non-heme ferritin was detected in our proteome, and its abundance was low across all treatments. It is possible that the application of targeted proteomics could better detect lower copy numbers (Saito et al., 2015).

### 4.4 Key warming and Fe response proteins

In both strains, heightened abundances of the Fe binding protein of an Fe(III) transport system (FutA/IdiA homologue) as well as Fe uptake porin proteins signify an enhancement in both active and passive transport into the cell in response to Fe limitation across both temperatures. Cyanobacteria are thought to utilize the FutABC iron uptake system to actively transport Fe from the periplasm into the cytoplasm, and FutA/IdiA homologues have been observed to be widely utilized in marine cyanobacteria and have been documented as biomarkers of Fe stress (Webb et al., 2001; Saito et al., 2014). The other two components of this Fe transport system, an inner membrane channel and an ATP-binding protein, were not detected in the proteome of either strain, except for coastal XM-24’s ATP-binding protein, which was measured at low and variable abundance, so was removed in filtering steps (see Methods). A recently growing body of work has pointed to the role of porins in passive uptake of free Fe ions (Jiang et al., 2015; Qiu et al., 2018). These small outer membrane protein channels allow for passive diffusion of small ions into the cell, but this flux is slow and unlikely on its own to supply enough Fe to the cell under Fe limitation. The potential importance of porins in Fe transport in Fe limited cells is supported by other studies (Qiu et al., 2021; Gilbert et al., 2022), but a comprehensive understanding of their place in cyanobacterial Fe transport systems remains unclear.

We observed interactive effects of warming and Fe limitation on abundances of different paralogs of ferredoxin that suggest their importance in the acclimation of each strain to these climate stressors. The Fe-S protein centers of ferredoxins allow them to carry electrons within multiple metabolic pathways, including photosynthesis, chlorophyll biosynthesis, and N assimilation (Cassier-Chauvat and Chauvat, 2014). The abundance of one paralog of ferredoxin, Ferredoxin 2Fe-2S, declined in response to Fe limitation for both strains and appears to be a sign of low Fe stress, except in coastal XM-24’s 30L treatment. Flavodoxin, which replaces ferredoxin under low Fe conditions in some phytoplankton (La Roche et al., 1996) was not present in the genomes of either strain in our study.

Both strains reduced abundances of ferredoxin-dependent components of the nitrate assimilation pathway in response to Fe limitation across all temperatures. Additionally, both strains demonstrated a synergistic reduction in urea metabolism proteins in response to simultaneous Fe limitation and warming. This shows that cells may respond to low Fe by decreasing their ability to use nitrate, since the assimilation of nitrate requires more Fe than reduced forms of N such as urea (Schoffman et al., 2016), while warming may interact with Fe limitation to additionally lower their capacity to utilize urea. Similarly, temperature has also been shown to interact with N source (i.e., nitrate and urea) on the growth success of a diatom species (Kelly et al., 2021). Alternatively, the decline in abundance of N assimilatory proteins could suggest a lower N quota under Fe limitation or an increased efficiency of urea assimilation at the higher temperature under Fe limitation (Li et al., 2019). In either case, the prevalence of urea as the main N source in coastal areas has been increasing due to rising input from anthropogenic runoff (Wang et al., 2020), so it is likely coastal and oceanic ecotypes will experience increasingly divergent N profiles in the future ocean.

Further, enzymes related to the amino acid glutamate, an essential molecule for N assimilation and metabolism, were significantly reduced under Fe limitation and its combination with warming (Supplementary Table S2). For example, glutamate synthase, a critical enzyme in the GS-GOGAT pathway, is the key regulatory point that assimilates N to synthesize glutamate (Muro-Pastor et al., 2005; Díez et al., 2023). The Fe-S center of this enzyme as well as its reliance on reducing power from ferredoxin and ATP from photosynthesis indicate various reasons for the observed decline in the concentration of glutamate synthase under Fe limitation in the oceanic strain. As glutamate serves as the main integrated N source, changes to concentrations of glutamate synthase would have cascading effects throughout the cell. Similarly, abundance of glutamate-cysteine ligase which catalyzes the formation of the stress response molecule glutathione significantly decreased in the coastal strain, possibly indicating an impaired ability to detect and respond to oxidative stress (Föller et al., 2013). Additionally, glutamyl-tRNA synthetase, which catalyzes the attachment of glutamate to its corresponding tRNA, significantly declined in abundance under Fe limitation and its interaction with warming in the oceanic strain. Reductions in abundance of multiple tRNA synthetases were observed in both strains, in agreement with a major slowdown of translation in response to the climate stressors.

Some paralogs of ferredoxin have also been studied for their role in detoxification of ROS under heat stress (Lin et al., 2013) or Cu limitation (Hippmann et al., 2017), as well as in overseeing Fe levels in the cell to control Fe homeostasis (Schorsch et al., 2018). One paralog of ferredoxin in our study synergistically increased in abundance in oceanic YX04-1 acclimated to the 30L treatment. We propose that this paralog of ferredoxin may aid in oxidative stress mitigation and contribute to oceanic YX04-1’s enhanced response to oxidative stress compared with coastal XM-24.

## 5 Conclusion

This study reveals the proteomic mechanisms behind *Synechococcus* acclimation to concurrent warming and Fe limitation. By comparing these responses between oceanic strain YX04-1 and coastal strain XM-24, we underscore how ecotypic diversity directs acclimation strategies and physiological outcomes.

We observed interactive effects between warming and Fe limitation on the physiology and proteomes of strains, whereby Fe limited YX04-1 was able to employ molecular strategies to partially mitigate the deleterious effects of warming on growth. These were driven by synergistic reductions in proteome size, modifications to photosynthetic organization, and enhanced oxidative stress responses. On the other hand, XM-24 did comparatively little to respond to these stressors. We speculate that in XM-24’s coastal environment, more abundant Fe has conditioned this strain to rely on ferritin for controlling its cellular Fe supply, rendering it less responsive to chronic Fe stress. Though coastal XM-24 was able to maintain higher growth rates in response to warming under replete Fe, it did not display a robust strategy to respond to the same thermal stress when combined with Fe limitation. We also delineated interesting responses of the cytochrome b6f complex, putative Fe uptake porins, chlorophyll binding proteins, and ferredoxins and suggest their potentially important role in the acclimation of *Synechococcus* to these climate stressors.

The South China Sea may experience reduced transport of nutrients such as Fe from the Pacific Ocean into the basin due to heat-induced changes in wind patterns (DeCarlo et al., 2017), supporting the relevance of this study. Comparable shifts in these stressors are projected to occur in other oceanic regions (Cheng et al., 2019; Tagliabue et al., 2020), and we suggest that our findings may also extend to other *Synechococcus* ecotypes under similar Fe regimes. However, effects of climate change on Fe-rich dust and terrestrial sources which also contribute Fe to varying regions of the South China Sea remain uncertain and require more studies (Hutchins and Boyd, 2016; Zhang et al., 2022).

The proteome analysis yielded key insights, but certain physiological trends remained unexplained, indicating that more sensitive analyses may be necessary to capture subtle changes in the metabolism of each strain. Additional differences in acclimation strategies between strains may be owed to post-translational or epigenetic modifications which were not accounted for by our methods. A complete understanding of the effects of interactions between multiple climate stressors on phytoplankton is crucial, and will require further efforts utilizing molecular analyses that additionally capture transcriptional and post-translational regulation to gain a more comprehensive picture of diverse *Synechococcus* metabolic strategies in the future ocean.

## Data availability statement

The genomic sequences of Synechococcus strains YX04-1 and XM-24 are available under GenBank accession numbers JAUONZ000000000 and QCWF01000000, respectively. Mass spectrometry data are deposited in the ProteomeXchange Consortium via the PRIDE partner repository with the identifier PXD045922. Code for data analysis and visualizations are available at https://github.com/cschiksnis/syn-proteome.

## Author contributions

CS: Conceptualization, Data curation, Formal analysis, Investigation, Methodology, Project administration, Visualization, Writing – original draft, Writing – review & editing. MX: Data curation, Investigation, Methodology, Writing – review & editing. MS: Data curation, Funding acquisition, Methodology, Resources, Software, Writing – review & editing. MM: Data curation, Methodology, Resources, Software, Writing – review & editing. DM: Data curation, Methodology, Resources, Software, Writing – review & editing. XB: Data curation, Methodology, Resources, Writing – review & editing. SJ: Funding acquisition, Methodology, Resources, Writing – review & editing. QZ: Data curation, Funding acquisition, Methodology, Resources, Writing – review & editing. NY: Methodology, Visualization, Writing – review & editing. FF: Conceptualization, Data curation, Funding acquisition, Methodology, Project administration, Resources, Writing – review & editing. DH: Conceptualization, Funding acquisition, Methodology, Project administration, Resources, Supervision, Writing – review & editing.

## Footnotes

^1^ https://www.naturalearthdata.com/
