# Supplementary material for: Proteomics analysis reveals differential acclimation of coastal and oceanic Synechococcus to climate warming and iron limitation
Source: Front Microbiol. 2024 Feb 20;15:1323499. doi: 10.3389/fmicb.2024.1323499 (PMC10912551; doi:10.3389/fmicb.2024.1323499)
Supplement: Supplementary file 4 [file Data_Sheet_1.DOCX]

***Supplementary Material***

1. **Supplementary Figures**

**Supplementary Figure 1. Map of *Synechococcus* isolation sites in the South China Sea.** Different color markers indicate origin of isolation for oceanic strain YX04-1 (green) and coastal strain XM-24 (pink), and relevant geographic features are shown in italicized text. The color gradient indicates bathymetry for depths less than 200 m (0 m), between 200 m and 1000 m (200 m) and at least 1000 m (1000 m).


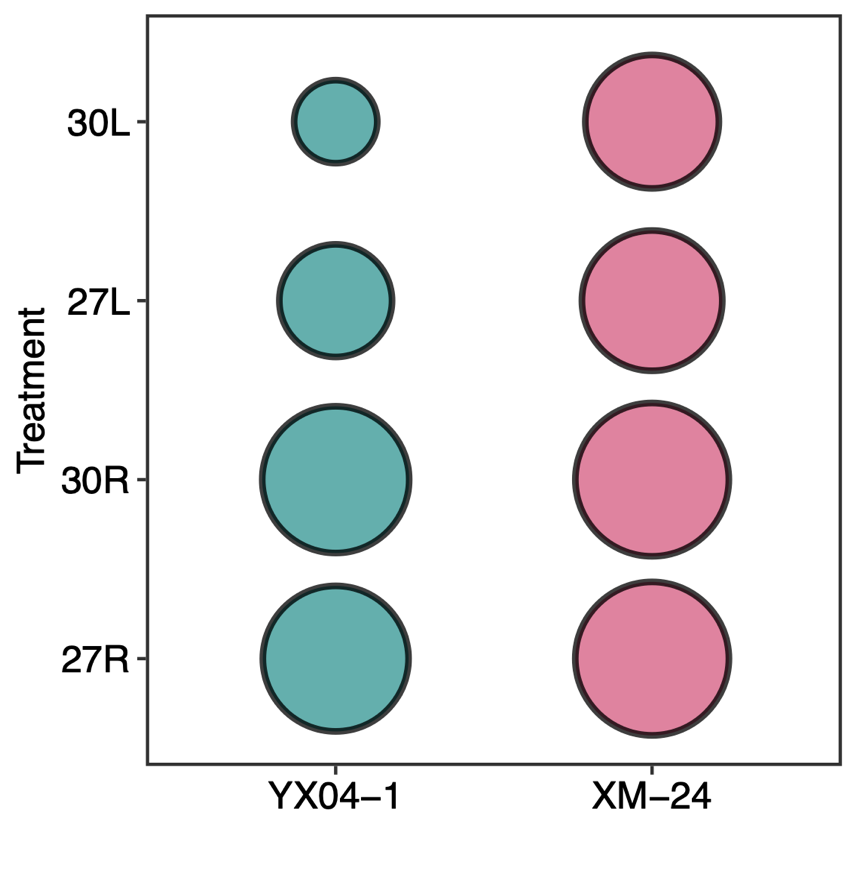


**Supplementary Figure 2. Proteome sizes across treatments and strains.** Size of each dot represents the size of the proteome in that treatment. Treatments are denoted on the y axis, with the number representing the temperature and the letter representing the Fe treatment (L, limited; R, replete). Strains are denoted on the x axis and by color.

**Supplementary Figure 3. Principal Component Analysis (PCA) of proteomes of *Synechococcus* strains (A) YX04-1 (oceanic) and (B) XM-24 (coastal) grown under 2 temperatures and 2 iron conditions.** Each point represents one replicate, and color and shape denote temperature and Fe condition, respectively.

**Supplementary Figure 4. Abundances of components of the cytochrome b6f complex of oceanic strain YX04-1 (left) and coastal strain XM-24 (right).** Components of the cytochrome b6f complex are listed on the righthand side of each row. Box plots represent treatment means of 3 replicates, and unique letters denote statistically significant means (within strains) for each protein. Statistical significance was calculated using a two-way ANOVA and Tukey post hoc testing (*p* < 0.05).

**Supplementary Figure 5. Abundances of N assimilation proteins for strain YX04-1 (left) and strain XM-24 (right).** Shortened names for ferredoxin-nitrate reductase, ferredoxin-nitrite reductase, and urease subunit alpha are listed on the righthand side of each row. Box plots represent treatment means of 3 replicates, and unique letters denote statistically significant means (within strains) for each protein. Statistical significance was calculated using a two-way ANOVA and Tukey post hoc testing (*p* < 0.05). Nitrate reductase was not detected in the proteome of YX04-1.

**Supplementary Figure 6. Average abundances of core photosystem (PS) proteins for oceanic YX04-1 (top) and coastal XM-24 (bottom).** Colors represent the individual protein, and proteins are grouped by either PSII or PSI (x axis bottom), and arranged by treatment (x axis top). The PSII:PSI ratio for each treatment is shown above each set of bars. Strain XM-24 had one extra PSII protein (PsbD, white) which was not detected in strain YX04-1, but this protein was not included in ratio calculations for consistency between strains. Error bars represent standard deviation of 3 experimental replicates.

**Supplementary Figure 7. Abundances of chlorophyll binding proteins for each strain.** Abbreviated names for photosystem II CP43 chlorophyll apoprotein and photosystem II CP47 chlorophyll apoprotein are listed on the righthand side of each row. Box plots represent treatment means of 3 replicates, and unique letters denote statistically significant means (within strains) for each protein. Statistical significance was calculated using a two-way ANOVA and Tukey post hoc testing (*p* < 0.05).

**2 Supplementary Note**

*isiA* was not found in the genomes of either strain, based on a nucleotide BLAST search that queried a database of *isiA* sequences against the genomes of each strain (Li et al., 2019). Our findings are consistent with recent genomic surveys that have revealed low *isiA* abundance in some *Synechococcus* ecotypes, especially in strains from the South China Sea (Chen et al., 2018; Li et al., 2019). Abundances of chlorophyll binding proteins, however, were elevated in both strains’ responses to Fe limitation, and some were synergistically enhanced in response to its combination with warming (**Supplementary Figure 7**). A homolog of IsiA (PsbC, CP43), increased in each strain’s 30L treatment, though this increase was significant only in YX04-1’s interaction scenario. CP47, also a PSII light harvesting protein, was also measured in elevated abundances in the 27L and 30L treatments for YX04-1, and increased in XM-24’s 30L treatment.

Heat stress has been found to cause aggregation of the light harvesting complexes of PSII in higher plants as a protective mechanism to dissipate excess excitation energy, which occurs due to reduced electron demand under decreased rates of CO_2_ fixation (Tang et al., 2007). We speculate that increases in these proteins may aid in photoprotection of both photosystems in our study. Their increased abundances specifically in response to Fe limitation at 30°C also suggests their unique role in the response to the combination of warming and Fe limitation.

**3 Supplementary Methods**

***Protein extraction***

Sample pellets were resuspended in protein extraction buffer (50mM HEPES pH 8.5, Boston BioProducts #BB-2082, 1% SDS in HPLC grade water) made with HPLC grade water (as are all reagents in this protocol). Sample resuspension volume varied depending on pellet size and solubility (400µL was aliquoted for processing and the remaining volume was stored at -20C). Resuspended samples were heated at 95˚C for 10 minutes and gently shaken at room temperature for 30 minutes. The protein extracts were centrifuged at 14100 x g (14500 rpm) for 20 min at room temperature. Supernatant was removed from pellet into a new tube. All tubes in further methods are ETOH washed.

***Protein reduction and alkylation***

50 units (2µL) of benzonase nuclease (Novagen #70746-3) was added to each 400µL extracted protein sample and incubated at 37˚C for 30 minutes. Samples were reduced by adding 20µL of 200mM DTT (Fisher #BP172-5) in 50mM HEPES pH 8.5 at 45˚C for 30 minutes. Samples were alkylated by adding 40µL of 400mM iodoacetamide (Acros #122270050) in HEPES pH 8.5 for 30 minutes at 24˚C, occasionally heating to 37˚C to prevent precipitation. The reaction was quenched by adding 40µL of 200mM DTT in 50mM HEPES pH 8.5.

***Protein clean up and digestion***

SpeedBead Magnetic Carboxylate Modified Particles (GE Healthcare #65152105050250 and #45152105050250) were prepared according to Hughes et al. (2014). 20µL (20µg/µL) of magnetic beads were added to 400µL of extracted protein sample. Samples were heated at 37˚C periodically to avoid precipitation. Samples were acidified to a pH of 2-3 by adding 50µL of 10% formic acid. 2X volume (1100µL) of acetonitrile was immediately added. Samples were incubated at 37˚C for 15 minutes and then at room temperature for 30 minutes. Samples were placed on a magnetic rack, incubated for 2 minutes, supernatant was removed and discarded. Samples were washed 2 times with 1400µL of 70% ETOH for 30 seconds on the magnetic rack. Supernatant was removed and discarded. 1400µL of acetonitrile was added to each sample for 30 seconds on the magnetic rack. Supernatant was removed and discarded. Samples air dried for approximately 4 minutes until acetonitrile had just evaporated. Samples were removed from the magnetic rack and beads were reconstituted in 90µL of 50mM HEPES pH 8.0. Purified protein was quantified with 2µL of sample in duplicate using the BCA method (Thermo Scientific Micro BCA Protein Assay Kit #23235). Standard curves were generated using albumin standard (Thermo Scientific #23210). Absorbance was measured on a Nanodrop ND-1000 spectrophotometer (Thermo Scientific). Trypsin (Promega #V5280) dissolved in HEPES pH 8.0 at a concentration of .5µg/µL was added to samples at a 1:25 trypsin to protein ratio and incubated at 37˚C overnight.

***Peptide recovery and preparation***

Acetonitrile was added to digested peptides at a concentration of ≥ 95% and incubated for 20 minutes at room temperature. Samples were then placed on the magnetic rack for 2 minutes and supernatant was removed and discarded. 1400µL of acetonitrile was added to samples on the magnetic rack for 15 seconds. Supernatant was removed and discarded. Samples air dried for approximately 4 minutes, just until acetonitrile was evaporated. Beads were reconstituted in 90µL of 2% DMSO and incubated off the rack at room temperature for ≥ 15 minutes. Samples were centrifuged slowly and briefly at 900 rcf to remove liquid from the tube walls. Samples were incubated on the magnetic rack for 15 minutes and supernatant containing peptides was transferred to a new ETOH washed 1.5ml microtube. This step was repeated to insure removal of all magnetic beads. 1% trifluoroacetic acid or 1% formic acid was added to samples for a final concentration of 0.1%. Peptides were quantified as described above. Samples with peptide concentrations > 1µg/µL were diluted in the corresponding buffer to a final concentration of 1µg/µL.

***Mass spectrometry***

Tryptic peptides were analyzed via liquid chromatography tandem mass spectrometry (LC/MS/MS) using a Michrom Advance HPLC system with reverse phase chromatography coupled to a Thermo Scientific Q-Exactive Orbitrap mass spectrometer with a Michrom Advance CaptiveSpray source. Each sample was concentrated onto a trap column (0.2 x 10 mm ID, 5 µm particle size, 120 Å pore size, C18 Reprosil-Gold, Dr. Maisch GmbH) and rinsed with 100 µL 0.1% formic acid, 2% acetonitrile (ACN), 97.9% water before gradient elution through a reverse phase C18 column (0.1 x 150 mm ID, 3 µm particle size, 120 Å pore size, C18 Reprosil-Gold, Dr. Maisch GmbH) at a flow rate of 500 nL/min. The chromatography consisted of a nonlinear 220 min gradient from 5% to 95% buffer B, where A was 0.1% formic acid in water and B was 0.1% formic acid in ACN (all solvents were Fisher Optima grade). The mass spectrometer monitored MS1 scans from 380 m/z to 1280 m/z at 70K resolution. MS2 scans were performed on the top 15 ions with an isolation window of 2.0 m/z and a 15 second exclusion time.

Mass spectra were searched against the translated transcriptomes using Proteome Discoverer’s SEQUEST HT algorithm (Thermo) with a fragment tolerance of 0.02 Da and parent tolerance of 10 ppm. Identification criteria consisted of a protein threshold of 1% FDR, peptide threshold of 0.1% FDR, and two minimum peptides when analyzed with Scaffold version 5.1.2 (Proteome Software, Inc.).

**4 Supplementary References**

Chen, H.-Y. S., Bandyopadhyay, A., and Pakrasi, H. B. (2018). Function, regulation and distribution of IsiA, a membrane-bound chlorophyll a-antenna protein in cyanobacteria. *Photosynthetica* 56, 322–333. doi: 10.1007/s11099-018-0787-7

Hughes, C. S., Foehr, S., Garfield, D. A., Furlong, E. E., Steinmetz, L. M., and Krijgsveld, J. (2014). Ultrasensitive proteome analysis using paramagnetic bead technology. *Molecular Systems Biology* 10: 757. doi: 10.15252/msb.20145625

Li, Q., Huisman, J., Bibby, T. S., and Jiao, N. (2019). Biogeography of Cyanobacterial *isiA* Genes and Their Link to Iron Availability in the Ocean. *Front. Microbiol.* 10. doi: 10.3389/fmicb.2019.00650
